# Altered functional connectivity of the nucleus tractus solitarii in patients with chronic cough after lung surgery: an rs‐fMRI study

**DOI:** 10.1111/1759-7714.15110

**Published:** 2023-09-17

**Authors:** Ming‐sheng Wu, Zheng‐wei Chen, Xiao Chen, Gao‐xiang Wang, Chun‐sheng Xu, Yong‐fu Zhu, Ming‐ran Xie

**Affiliations:** ^1^ Division of Life Sciences and Medicine University of Science and Technology of China Hefei China; ^2^ Department of Thoracic Surgery, The First Affiliated Hospital of USTC, Division of Life Sciences and Medicine University of Science and Technology of China Hefei China; ^3^ Medical Imaging Center The First Affiliated Hospital of Anhui University of Chinese Medicine Hefei China; ^4^ The First Department of Oncology The First Affiliated Hospital of Anhui University of Chinese Medicine Hefei China

**Keywords:** anxiety, cough, functional connectivity, nucleus tractus solitarii, resting‐state functional magnetic resonance imaging

## Abstract

**Background:**

To explore the altered functional connectivity (FC) of the nucleus tractus solitarii (NTS) in patients with chronic cough after lung surgery using resting‐state functional magnetic resonance imaging (rs‐fMRI), and the association between abnormal FC and clinical scale scores.

**Methods:**

A total of 22 patients with chronic cough after lung surgery and 22 healthy controls were included. Visual analog scale (VAS), Mandarin Chinese version of the Leicester Cough Questionnaire (LCQ‐MC), and Hamilton anxiety rating scale (HAMA) scores were assessed, and rs‐fMRI data were collected. The FC analysis was performed using the NTS as the seed point, and FC values with all voxels in the whole brain were calculated. A two‐sample *t*‐test was used to compare FC differences between the two groups. The FC values of brain regions with differences were extracted and correlated with clinical scale scores.

**Results:**

In comparison to healthy controls, FC values in the NTS and anterior cingulate cortex(ACC) were reduced in patients with chronic cough after lung surgery (GRF correction, p‐voxel < 0.005, p‐cluster < 0.05) which were positively correlated with LCQ‐MC scores (*r* = 0.534, *p* = 0.011), but with VAS (*r* = −0.500, *p* = 0.018), HAMA (*r* = −0.713, *p* < 0.001) scores were negatively correlated.

**Conclusions:**

Reduced FC of the NTS with ACC may be associated with cough hypersensitivity and may contribute to anxiety in patients with chronic cough after lung surgery.

## Graphical abstract

Compared with the healthy group, the functional connectivity (FC) between the nucleus tractus solitarii (NTS) and anterior cingulate cortex (ACC) was decreased in patients with chronic cough. Using the NTS as the seed point, the left ACC is the brain region with lower whole‐brain FC in chronic cough patients than in healthy people, and blue represents the FC weakness.

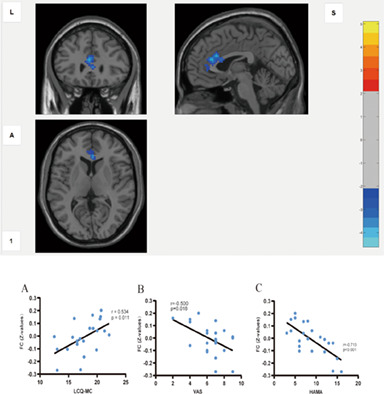

## INTRODUCTION

Chronic cough is the most common complication after lung surgery, which seriously affects patients' quality of life.^1^, ^2^, ^3^ Neuroimaging studies, particularly resting‐state functional magnetic resonance imaging (rs‐fMRI) studies, have revealed that cough is not only an airway‐protective behavior caused by peripheral airway stimulation but also involves multiple brain networks interacting abnormally.^4^, ^5^, ^6^, ^7^ Airway sensory information is transmitted via vagal sensory fibers to termination sites within the dorsal brainstem, primarily the nucleus tractus solitarii (NTS) and the paracentral trigeminal nucleus, before projecting to higher nociceptors.^8^, ^9^ Changes in the connectivity network of brainstem nuclei associated with cough, on the other hand, are largely unknown. Neurophysiological and pharmacological studies have demonstrated that the NTS plays an important regulatory role in the central reflex of cough, that the NTS is involved in regulating cough excitability and cough rhythm^10^ and over‐activation of NTS neurons may be associated with chronic cough.^11^ Therefore, further analysis of the abnormal connections between the NTS region and other brain regions would help to better understand the pathogenesis of cough. In this study, we used the functional connectivity (FC) method based on the fMRI technique to analyze the changes in functional connectivity between the NTS and the whole brain in patients with chronic cough after lung surgery and to explore the correlation between FC changes and clinical symptoms to improve our understanding of the central neural mechanisms of chronic cough.

## METHODS

### Patient selection

A total of 22 patients (five males and 18 females) with chronic cough after lung cancer surgery attending the Department of Thoracic Surgery, The First Affiliated Hospital of the University of Science and Technology of China were prospectively and consecutively recruited into this study between November 2021 and May 2022. Patients were included if they: (1) were diagnosed with NSCLC according to histopathology, (2) underwent lobectomy or segmental lung resection + mediastinal lymph node dissection, (3) had cough duration ≥8 weeks, (4) had no other neurological underlying disease, (5) were right‐handers, (6) had signed informed consent, and excluded if they: (1) had an acute respiratory disease within 1 month, (2) had pneumonia on a positive chest X‐ray, (3) had contraindications to MRI scanning, or (4) had structural brain alterations revealed by MRI.

Noncough healthy people matched for age and sex were also enrolled as controls. The inclusion criteria for 22 healthy controls (HC) were as follows:(1) no symptoms of cough, (2) conventional MRI of the head was normal, (3) absence of psychiatric and neurological disorders, (4) denied a history of drug or alcohol abuse and (5) signed informed consent.

The Ethics Committee of the First Hospital of the University of Science and Technology of China approved this study based on the Declaration of Helsinki. Before the study, each subject provided written informed consent.

### Clinical assessment of participants

Before the MRI scan, the investigators collected clinical information from the subjects, including gender, age, duration, and so on. The cough was assessed by a visual analog scale (VAS) and the Mandarin Chinese version of the Leicester Cough Questionnaire (LCQ‐MC). All enrolled subjects received the VAS and LCQ‐MC. The anxiety of patients was assessed using the Hamilton anxiety rating scale (HAMA).

### Functional magnetic resonance imaging parameters

All data acquisition was performed on a 3.0 T magnetic resonance system (Siemens) at the Medical Imaging Center of the First Affiliated Hospital of Anhui University of Traditional Chinese Medicine. Before scanning, tight and comfortable foam padding and earplugs were used to reduce head moving and noise in the scanner separately. T2‐weighted imaging was used to rule out other organic lesions in the brain, the following parameters: repetition time (TR) = 3457 ms, echo time (TE) = 82 ms, field of view (FOV) = 240 × 240 mm, slice thickness = 5 mm, interslice gap = 1.5 mm, and 24 layers scanned. T1 structural images were scanned with TR = 2000 ms, TE = 3.2 ms, flip angle (FA) = 12°, matrix =256 × 256, FOV = 256 × 256 mm, layer thickness = 1 mm, 166 layers scanned. Resting state fMRI imaging data collection with gradient recalled echo‐planar imaging (GRE‐EPI), same scan orientation and number of layers as 2D anatomical image, TR = 2000 ms, TE = 35 ms, FA = 90°, layer thickness = 3.0 mm, layer spacing = 1 mm, 40 layers scanned, FOV = 220 × 220 mm, resolution = 64 × 64, 200 time points acquired.

### Data preprocessing

Preprocessing of rs‐fMRI images and structural images was performed using the SPM12 (http://www.fil.ion.ucl.ac.uk/spm) and DPABI packages^12^ (http://rfmri.org/DPABI) running on Matlab R2019b. To exclude instability of the MRI instrument and subject discomfort at the start of the scan session, we excluded the first 10 time points of data for each subject. The remaining 190 time points were then subjected to time‐layer correction and head motion correction. Subjects were excluded if they had a maximum translation >2 mm or rotation >2° in any direction. Then we used the anterior–posterior union as the origin of the high‐resolution T1‐weighted images, aligned the functional images of all subjects with the structural images, and segmented the images into white matter, gray matter, and cerebrospinal fluid. The images were normalized to Montreal Neurological Institute (MNI) space and resampled at a size of 3 × 3 × 3 mm. A 6 × 6 × 6 mm smoothing kernel was then chosen to spatially smooth the preprocessed data, and a general linear model was used to regress the head movement, cerebrospinal fluid signals, and white matter signals. The preprocessed images were filtered using band‐pass filters in the frequency range of 0.01–0.1 Hz to remove low‐ and high‐frequency physiological signals.

### Functional connectivity analysis

Based on previous studies in the literature,^13^ the NTS (MNI: 0, −43, −54) was selected as the seed point, and the radius was set to 4 mm. The mean time signals of these spherical seed areas were extracted, the time series of the seed areas were correlated with the time series of all voxels in the whole brain, and the correlation coefficients were converted to z values using Fisher transformation to enhance normality.

### Statistical analysis

The statistical analysis was performed using SPSS 26.0 (SPSS). The mean ± standard deviation was used for normally distributed data, and independent sample *t*‐tests were used to compare groups. *p* < 0.05 was considered a statistically significant difference.

Age, gender, and head movement parameters were used as covariates, and the two groups of FC values were subjected to an independent samples *t*‐test using DPABI. The voxel‐level significance threshold was set at *p* < 0.005 combined with a cluster‐level threshold of *p* < 0.05, and corrected for multiple comparisons using the Gaussian random field (GRF). The *z*‐values for the differential brain regions were extracted and correlated with clinical scale scores using SPSS 26.0. A Pearson correlation analysis was performed with the clinical scale scores using SPSS 26.0, with *p* < 0.05 being a statistically significant difference; |*r*| the larger the value, the stronger the correlation.

## RESULTS

### Demographic and clinical characteristics

The demographics and clinical characteristics of the subjects are shown in Table 1. No significant differences in age (*p* = 0.497) or gender (*p* = 0.876) were found between the chronic cough and HC groups.

**TABLE 1:** Demographic and clinical characteristics of chronic cough and HC.

Variables	Chronic cough (*n* = 22)	HC (*n* = 22)	*T*‐value	*p*‐value
Age (year, mean ± SD)	57.64 ± 11.47	55.32 ± 10.42	−0.685	0.497
Gender (male/female)	4/18	7/15	NA	0.296
Leigh hand (right/left)	22/0	22/0	NA	‐
Duration of illness (weeks)	9.5 ± 1.47	‐	NA	‐
VAS score (mean ± SD)	6.35 ± 1.77	‐	NA	‐
LCQ‐MC score	17.71 ± 2.72	‐	NA	‐
HAMA	8.86 ± 3.96	‐	NA	‐

Abbreviations: HAMA, Hamilton anxiety rating scale; HC, healthy controls; LCQ‐MC, Mandarin Chinese version of the Leicester cough questionnaire; VAS, visual analog scale.

### Functional connectivity results

**FIGURE 1:**
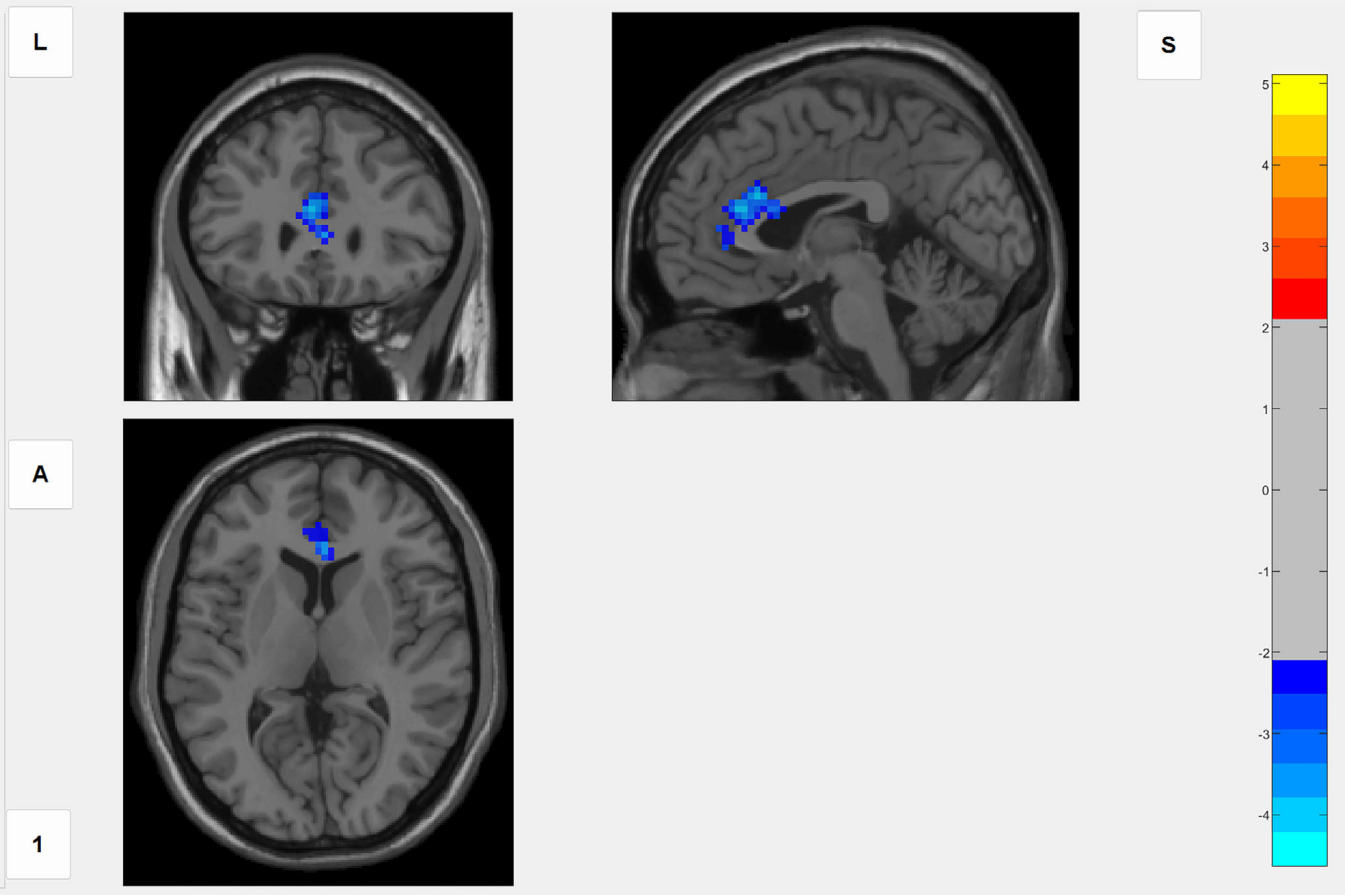
Compared with the healthy group, the functional connectivity (FC) between nucleus tractus solitarii (NTS) and anterior cingulate cortex (ACC) was decreased in patients with chronic cough (GRF correction, p‐voxel < 0.005, p‐cluster < 0.05). Using the NTS as the seed point, the left ACC is the brain region with lower whole‐brain FC in chronic cough patients than in healthy people, and blue represents the FC weakness.

In comparison to the HC group, the chronic cough group only had a weakened functional connectivity between the NTS and the left anterior cingulate cortex (ACC) (Figure 1).

### Correlations with clinical characteristics

**FIGURE 2:**
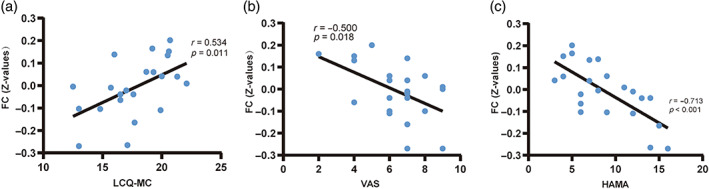
Correlation analysis between the scale scores and functional connectivity (FC) values in the nucleus tractus solitarii and anterior cingulate cortex. (a) The Mandarin Chinese version of the Leicester Cough Questionnaire (LCQ‐MC) scores showed a positive correlation with FC values (*r* = 0.534, *p* = 0.011). (b) The visual analog scale (VAS) scores showed a negative correlation with FC values (*r* = −0.500, *p* = 0.018). (c) The anxiety scores showed a negative correlation with FC values (*r* = −0.713, *p* < 0.001).

Pearson correlation analysis showed that LCQ‐MC (*r* = 0.534) scores were significantly positively correlated with FC values in NTS‐left ACC, and VAS (*r* = −0.500) and HAMA (*r* = −0.713) were significantly negatively correlated with FC values in NTS‐left ACC (Figure 2).

## DISCUSSION

The mechanisms of chronic cough after lung surgery include: (1) local chronic inflammatory changes caused by surgical damage to lung tissue and peribronchial nerves and some glands; (2) physical changes to the small airways after surgery, such as poor ventilation due to local torsion into angles; (3) chronic irritation from foreign bodies such as surgical scars and sutures in the airways; and (4) local pleurisy and pleural effusion.^14^, ^15^ The common denominator of the above factors is chronic irritation of the distal airways and lung parenchyma. After stimulation of the sensory nerve endings, nerve impulses are transmitted along the vagus nerve to the cough center in the brainstem, and the signals are integrated and transmitted along the efferent nerve to the effector, ultimately resulting in the cough action.^16^

Regarding the central mechanism of cough, the current view is that the medulla oblongata is involved in the regulation of the central cough reflex.^17^ When the body is in a state of stress or chronic inflammatory stimulation, the neurons of the NTS can show neuroplasticity changes.^18^, ^19^ In the study by Mazzone et al.,^20^ 10 normal volunteers were separately assigned to inhale capsaicin or inhale saline (control group), and each group's cough impulse was recorded and functional brain imaging was performed. The results showed that capsaicin could stably induce the cough impulse and was associated with the activation of the cerebral cortex, suggesting that the cortical neural network may be involved in the regulation of human cough. Joad et al.^21^ exposed guinea pigs to cigarettes (1 mg/m3, 6 h/day, 5 days/week) and their cough sensitivity to citric acid increased significantly after 5 weeks of exposure. At week 6, the substance P antagonist SR140333 (neurokinin‐1 receptor antagonist) was injected. The results showed that SR140333 could significantly inhibit the cough sensitivity of guinea pigs exposed to cigarettes, suggesting that the NTS may increase cough sensitivity by releasing substance P.

In this study, we used rs‐fMRI to assess changes in functional connectivity between the NTS and other brain areas in patients with chronic cough after lung surgery. The results showed that functional connectivity between NTS and left ACC was reduced in patients with chronic cough compared to the HC group. The change in functional connectivity pattern was significantly positively correlated with LCQ‐MC score and negatively correlated with VAS and HAMA score, suggesting that the decrease in functional connectivity between NTS and left ACC may be related to the occurrence of cough hypersensitivity and anxiety in patients with chronic cough. To our knowledge, this is the first study to observe differences in functional brain activity in chronic cough after lung surgery cough patients.

The ACC is a cerebral gyrus located between the medial pocket sulcus and corpus callosum in the cerebral hemisphere and belongs to the cortical part of the limbic system, which is involved in a variety of higher functions, including nociception, reward, decision‐making, and motivation.^22^, ^23^, ^24^ ACC is involved in constituting the respiratory regulatory network^25^ and mediates a compensatory physiological response to cough stimuli, and inhalation of capsaicin in healthy subjects induced a significant increase in ACC blood oxygen levels. In healthy subjects, inhalation of capsaicin causes a significant increase in ACC blood oxygen levels.^26^ In contrast, blood oxygen levels in the ACC region are reduced in patients with chronic cough,^5^, ^27^ while recent studies have found individuals with chronic cough have a smaller volume of the anterior cingulate cortex.^27^ Meanwhile, our results showed a significant positive correlation between the FC values of the NTS‐ACC and LCQ‐MC scores and a negative correlation with the VAS, which we speculate may be due to plastic changes in the ACC as a result of chronic cough after lung surgery, with a decrease in its own function and weakened functional connectivity with other nuclei, resulting in the loss of cough suppression and leading to the generation of cough hypersensitivity responses.^27^

Furthermore, the ACC is also involved in the cognitive and emotional processing of perceptual signals,^28^ and structural changes in this region may negatively affect aspects of cough emotional processing and perceived cough severity in patients with chronic cough after lung surgery,^27^, ^28^ which is consistent with our findings. Our study found higher HAMA anxiety scores in patients with lower NTS‐ACC functional connectivity, suggesting that activating the ACC may reduce anxiety symptoms in chronic cough patients.

Nevertheless, there are some limitations to this study. First, the sample size was small and the age range was large. Second, only a single MNI coordinate was used to select the seed area. Third, the voxel threshold level which was set at *p* < 0.005 in this study had weak statistical validity, and a voxel threshold level set at *p* < 0.001 would have been more reasonable.

In summary, this study analyzed the altered functional connectivity of the NTS in patients with chronic cough after lung surgery. The results showed, compared to healthy controls, the reduced FC of the NTS to ACC was associated with hypersensitivity to cough and may contribute to anxiety in patients with chronic cough compared to healthy controls. This finding further validates the important role of NTS in chronic cough and provides new ideas for the selection of therapeutic targets for chronic cough after lung surgery.

## AUTHOR CONTRIBUTIONS

Conception and design: Ming‐sheng Wu and Ming‐Ran Xie. Administrative support: Ming‐Ran Xie. Provision of study materials or patients: Yong‐fu Zhu and Ming‐Ran Xie. Collection and assembly of data: Ming‐sheng Wu, Zheng‐wei Chen and Xiao Chen. Data analysis and interpretation: Ming‐sheng Wu, Chun‐sheng Xu and Ming‐Ran Xie. Manuscript writing: All authors. Final approval of manuscript: All authors.

## CONFLICT OF INTEREST STATEMENT

No authors report any conflict of interest.
